# Reconfigurable Antenna Array Direction Finding System Based on a Fast Search Algorithm

**DOI:** 10.3390/s21144729

**Published:** 2021-07-10

**Authors:** Chunxi Liu, Dongliang Peng, Zhikun Chen, Yong Wu, Binan Wang

**Affiliations:** School of Automation, Hangzhou Dianzi University, Hangzhou 310018, China; lcxhdu@hdu.edu.cn (C.L.); dlpeng@hdu.edu.cn (D.P.); wuyong@hdu.edu.cn (Y.W.); wba1998@hdu.edu.cn (B.W.)

**Keywords:** reconfigurable antenna array, direction of arrival (DOA) estimation, microwave switches, fast DOA search algorithm

## Abstract

In a traditional antenna array direction finding system, all the antenna sensors need to work or shut down at the same time, which often leads to signal crosstalk, signal distortion, and other electromagnetic compatibility problems. In addition, the direction-finding algorithm in a traditional system needs a tremendous spectral search, which consumes considerable time. To compensate for these deficiencies, a reconfigurable antenna array direction finding system is established in this paper. This system can dynamically load part or all of the antennas through microwave switches (such as a PIN diode) and conduct a fast direction of arrival (DOA) search. First, the hardware structure of the reconfigurable antenna is constructed. Then, based on the conventional spatial domain search algorithm, an improved transform domain (TD) search algorithm is proposed. The effectiveness of the system has been proven by real experiments, and the advantage of the system has been verified by detailed simulations.

## 1. Introduction

With the rapid development of information technology, wireless transmission systems, such as mobile communications, radar, and unmanned aerial vehicles (UAV), have become widely used. As such, antenna technology has become more important, as has array antenna direction finding, which is an important branch of antenna technology [1,2,3,4,5].

A complete antenna array direction finding system consists of two parts, namely, the hardware structure of the antenna and a direction-finding algorithm. When a traditional antenna array system works, all the antennas need to run or shut down at the same time [6,7]. However, when the number of incident sources is small, only part of the antennas is needed to obtain an accurate direction of arrival (DOA). Moreover, if there are too many antennas working at the same time, the system will face intractable electromagnetic compatibility problems, such as signal crosstalk and signal distortion, which reduce the communication quality of the whole system [8,9,10]. To solve these problems, D. Schaubert proposed the concept of a reconfigurable antenna array system [11]. A reconfigurable antenna system can dynamically control the working state of the antenna according to the needs of a given environment [12,13,14]. When the number of target signals is small, the system will turn off some antennas. The system not only reduces resource consumption, but also effectively reduces electromagnetic compatibility between antennas [15,16,17]. In engineering applications, adjustable devices (such as microwave switch, variable capacitor, liquid metal material, or graphene) are usually loaded on the antenna surface, and then the current distribution on the antenna surface can be changed by controlling these adjustable devices, allowing the antenna state to be reconfigured [18,19,20].

Traditional antenna array direction finding technology mostly uses a method based on amplitude or phases to judge the direction of arrival; this method has low accuracy and is easily affected by noise [21,22]. With the development of technology, numerous subspace-based methods such as multiple signal classification (MUSIC) [23], maximum-likelihood [24], subspace fitting [25], and estimation of signal parameters via rotational invariance techniques (ESPRIT) [26] have attracted considerable attentions. Among these algorithms, the ESPRIT algorithm offering a much faster computing speed than the other beamformers is one of the most popular techniques. Unfortunately, the array geometry in ESPRIT is required to be shift-invariant [27]. Compared with ESPRIT algorithm, the MUSIC algorithm has no dependence on array structure and can offer higher accuracy [28]. However, as the conventional MUSIC involves a tremendous computation burden, it is prohibitively expensive when real-time processing is required. The high computational complexity of the MUSIC algorithm is mainly caused by a spectral search step. To avoid an exhaustive search over the whole angle space, a reduced-dimension MUSIC algorithm (RD-MUSIC) was proposed [29,30]; this algorithm can avoid high computational costs within a multi-dimensional search algorithm. However, this algorithm not only reduces the amount of computation but also reduces the accuracy of parameter estimation. To solve this problem, the concept of transform domain (TD) was proposed [31]. The authors in [31] transformed the received signal into the coarray domain and then iteratively corrected the phase offset between the coarray data and the presumed model caused by angle biases, according to a closed-form formula. However, this method is only applicable to a uniform liner array, which means that the method is not universal. Nevertheless, it is a concept worth learning and developing.

In this paper, a reconfigurable antenna array direction finding system is proposed, including both hardware and an algorithm. The hardware of the reconfigurable antenna is a double-layer structure: the upper layer is a radiation unit, which is printed on Rogers 5880; the lower layer comprises the matching circuit and the floor, both of which are printed on the front and back sides of the Rogers 4350B dielectric substrate. On the other hand, the essence of the proposed algorithm is to substitute the intersection of a transformed noise-like subspace cluster for the original noise subspace of the standard MUSIC to construct a new spatial spectrum. We have deployed the system in an actual environment and have carried out numerous tests. The test results show that the system can accurately estimate the DOA of the incident signal. Theoretical performance analysis on the root-mean-squared error (RMSE) and numerical simulations demonstrate that the proposed algorithm has higher accuracy and lower computational complexity than the MUSIC and RD-MUSIC algorithms.

## 2. Reconfigurable Antenna and Array Signal Model

### 2.1. Reconfigurable Antenna

**Figure 1:**
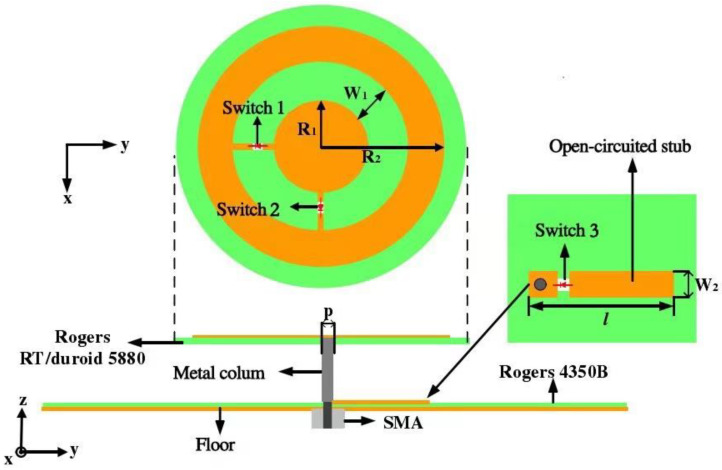
Reconfigurable antenna.

On the basis of [32], a reconfigurable antenna is proposed, the hardware of which is shown in Figure 1. The antenna mainly consists of two parts: (1) the upper layer is a radiation unit, which is printed on Rogers 5880. The dielectric constant of the dielectric substrate is 2.2, the dielectric loss angle is 0.009, the radius of the dielectric plate is 30 mm, and the thickness is 0.8 mm. The radiation element consists of an inner circle with radius $R_{1}$ and an outer ring with radius $R_{2}$. The distance between the inner circle and the outer ring is $W_{1}$. (2) The lower layer comprises the matching circuit and the floor, both of which are printed on the front and back sides of the Rogers 4350B dielectric substrate. The radius of the dielectric substrate is 70 mm, and the thickness is 0.8 mm. The upper and lower structures are connected by metal columns with a diameter of 1 mm. The antenna uses three PIN diodes, two of which are loaded on the gap between the inner circle and the outer ring of the radiation unit. The on–off state of the PIN diodes is controlled by the bias circuit to realize the reconfiguration of the antenna pattern. Another PIN diode is loaded on the matching circuit of the lower layer, and the on–off state of the diode is used to control the antenna turning on and off. When the reconfigurable antenna is applied to the direction-finding system, the system can dynamically control the working state of each antenna and determine the number of working antennas according to the number of signal sources.

### 2.2. Array Signal Model

**Figure 2:**
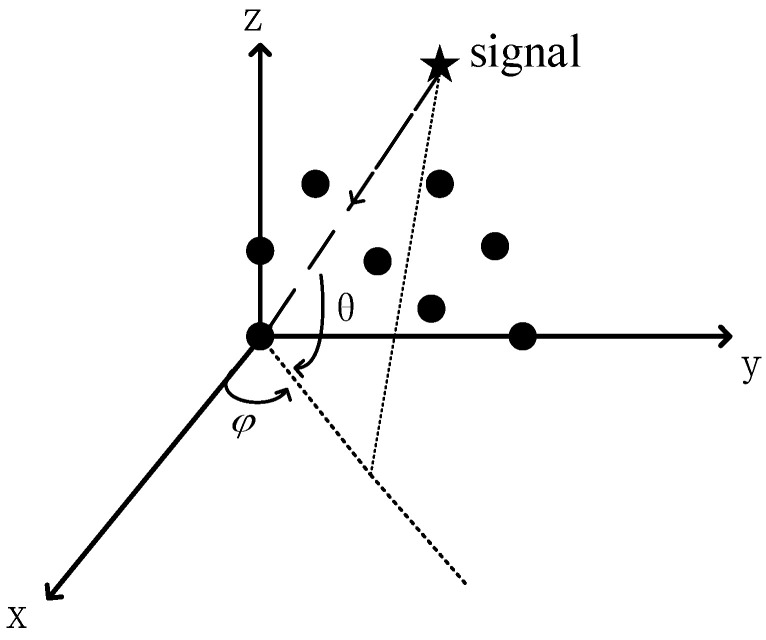
Reconfigurable antenna array.

Consider a reconfigurable antenna array composed of *M* antennas, as shown in Figure 2. Their positions are arbitrarily distributed on the YOZ plane, and the position coordinates of the antennas are given by $\left(0,\;y_{m},\;z_{m}\right),\;m=1,2,\cdots ,M$.

The black dots represent the antennas. We define the DOA angle as (θ,φ). θ is defined as the intersection angle between the signal and the projection of the signal onto the XOY plane, while φ is defined as the angle between the projection of the signal onto the XOY plane and the positive direction of the X-axis. Here, the value ranges of θ and φ are both [−π/2, π/2]. We assume that the number of incident sources is known and that there are K distinct uncorrelated signals coming from directions of $\left(\theta_{k},\varphi_{k}\right),k=1,2,\cdot \cdot \cdot ,K$, where $\theta_{k}$ and $\varphi_{k}$ are the spatial angles of the kth signal, respectively. The data received by a snapshot of the array can be expressed as:

(1) x(t)=A(θ,φ)s(t)+n(t)

where $x\left(t\right)≜{\left[x_{1}\left(t\right),x_{2}\left(t\right),\cdots ,x_{M}\left(t\right)\right]}^{T}$ is the M×1 dimensional array receiving data, $s\left(t\right)≜{\left[s_{1}\left(t\right),s_{2}\left(t\right),\cdots ,s_{K}\left(t\right)\right]}^{T}$ is the signal sampling data, and n(t) is the additive white Gaussian noise matrix with the same dimension as x(t). The array steering matrix $A\left(\theta ,\varphi \right)≜\left[a\left(\theta_{1},\varphi_{1}\right),a\left(\theta_{2},\varphi_{2}\right),\cdots ,a\left(\theta_{K},\varphi_{K}\right)\right]$ and the steering vector $a\left(\theta_{k},\varphi_{k}\right)$ can be expressed as:

(2) $$a\left(\theta_{k},\varphi_{k}\right)≜\left[e^{j\beta_{k,1}},e^{j\beta_{k,2}},\cdots ,e^{j\beta_{k,M}}\right]$$

where $e^{j\beta_{k,m}}$ is the phase difference of the kth signal of the mth element relative to the reference array element. Assume that λ is the signal wavelength; then, $\beta_{k,m}$ is given by:

(3) $$\beta_{k,m}=\frac{2\pi }{\lambda }\left(y_{m}\cos\theta_{k}\sin\varphi_{k}+z_{m}\sin\theta_{k}\right)$$

The process of estimating the 2D spatial angle of the K signals, based on the signal model constructed by Equation (1), is called spatial spectral estimation.

When the number of target signals is small, in order to reduce the loss of system resources, we can consider using only part of the antennas. However, the traditional array system needs to reset the whole system when modifying the antenna. In addition, the traditional algorithm uses the spacing of array elements to establish the steering vector, which is not flexible enough when adding or deleting antennas. Compared with a traditional system, our system does not need to reset the whole system when adjusting the antenna, and it can control the working state of each antenna online through the PIN diode. On the other hand, we use element coordinates instead of element spacing to establish the steering vector. When some antennas are offline, we only need to delete the corresponding coordinates in the model to continue the DOA estimation. This process achieves the goal of online reconfiguration.

## 3. Proposed Algorithm

### 3.1. Principle of the Traditional MUSIC Algorithm

In practical application, according to the received data from *P* snapshots, the spatial correlation is estimated using a time average, and the covariance matrix of the array output is obtained by:

(4) $${\hat{R}}_{XX}=\frac{1}{P}XX^{H}$$

where X is the received data matrix of *P* snapshots. After performing an eigenvalue decomposition, we can derive the following result:

(5) $${\hat{R}}_{XX}=U_{S}D_{S}U_{S}{}^{H}+U_{N}D_{N}U_{N}{}^{H}$$

where $U_{S}$ is the signal subspace composed of eigenvectors corresponding to *K* larger eigenvalues and $U_{N}$ is the noise subspace composed of eigenvectors corresponding to M−K smaller eigenvalues. Accordingly, we can see that the signal subspace and the space formed by the steering vector of the array are the same, and the steering vector space of the array and the noise subspace are orthogonal to each other [33]. This orthogonal relation can be expressed as:

(6) $$a^{H}\left(\theta ,\varphi \right)U_{N}=O$$

where the symbol ‘H’ represents the conjugate transpose operation, a(θ,φ) is the M×1 steering vector, $U_{N}\in {\mathbb{C}}^{M\times M-K}$ is the noise space, and O is the 1×M−K zero vector.

Considering that the length of the actual received data matrix is limited, and that noise is mixed in the actual received data matrix, a(θ,φ) and $U_{N}$ are not completely orthogonal. In other words, Equation (6) is not completely valid. The DOA parameters are estimated through the minimum optimization search, so the two-dimensional joint spectral function is defined as

(7) $$P_{MUSIC}\left(\theta ,\varphi \right)=\frac{1}{a^{H}\left(\theta ,\varphi \right)U_{N}U_{N}{}^{H}a\left(\theta ,\varphi \right)}$$

Theoretically, the DOA estimation in Formula (7) can be estimated by bringing a 2D search to bear on the ranges of all parameters; however, this is computationally exhaustive.

### 3.2. The Proposed Algorithm

When running the traditional MUSIC algorithm, an extreme value test should be conducted for each point in the spatial spectrum. With the improvement in search accuracy, the number of spectral points is increased further, leading to a sharp increase in the running time of the algorithm. If we can find a way to compress the extremum search range, then the speed of DOA estimation can be improved. From this analysis, the following transformation is considered:

(8) $$\left\{ \begin{array}{l} u=\cos\theta \sin\varphi \\ v=\sin\theta \end{array}\right..$$

Combining Equation (3) and Equation (8) leads to

(9) $$\beta_{k,m}=\frac{2\pi }{\lambda }\left(y_{m}u_{k}+z_{m}v_{k}\right).$$

Equation (8) shows that, for a given spatial domain (θ,φ), there is a unique transform domain (u,v) that corresponds to it. By contrast, Equation (8) also can be expressed as

(10) $$\left\{ \begin{array}{l} \theta_{1}=\sin^{-1}\left(v\right)\\ \varphi_{1}=\sin^{-1}\left(\frac{u}{\sqrt{1-v^{2}}}\right) \end{array}\right.\;\mathrm{or}\;\left\{ \begin{array}{l} \theta_{2}=\sin^{-1}\left(v\right)\\ \varphi_{2}=\pi -\sin^{-1}\left(\frac{u}{\sqrt{1-v^{2}}}\right) \end{array}\right.$$

Equation (10) shows that, for a given transform domain angle (u,v), there are two spatial angles (θ,φ) and (θ,π−φ) that correspond to it. Using Equation (6) to judge (θ,φ) and (θ,π−φ), the true angle of arrival can be obtained. Moreover, combining Equation (2) and Equation (9) leads to

(11) $$a\left(u,v\right)=\left[\begin{array}{l} e^{j2\pi \left(y_{1}u+z_{1}v\right)/\lambda }\\ e^{j2\pi \left(y_{2}u+z_{2}v\right)/\lambda }\\ \vdots \\ e^{j2\pi \left(y_{M}u+z_{M}v\right)/\lambda } \end{array}\right]={\left[\begin{array}{l} e^{j2\pi \left(y_{1}\left(-u\right)+z_{1}\left(-v\right)\right)/\lambda }\\ e^{j2\pi \left(y_{2}\left(-u\right)+z_{2}\left(-v\right)\right)/\lambda }\\ \vdots \\ e^{j2\pi \left(y_{M}\left(-u\right)+z_{M}\left(-v\right)\right)/\lambda } \end{array}\right]}^{*}=a^{*}\left(-u,-v\right)$$

where * represents the conjugate operation. The steering vector a(θ,ϕ) and the TD steering vector a(u,v) are clearly equivalent. Furthermore, according to Equation (6) and Equation (11), we can deduce that

(12) $$\left\{ \begin{array}{l} a^{H}\left(u,v\right)U_{N}=O_{M\times 1}\\ {\left[a^{*}\left(u,v\right)\right]}^{H}U_{N}{}^{*}=a^{H}\left(-u,-v\right)U_{N}{}^{*}=O_{M\times 1} \end{array}\right..$$

In Equation (12), we call (−u,−v) the symmetric signal source of (u,v), and the steering vector corresponding to the real signal source (u,v) is orthogonal to the noise subspace $U_{N}$, whereas the steering vector corresponding to the symmetric signal source (−u,−v) is orthogonal to the conjugate noise subspace $U_{N}{}^{*}$. If we replace the noise space in the MUSIC algorithm with the intersection space of $U_{N}$ and $U_{N}{}^{*}$, as the intersection space is orthogonal to the real steering vector and the symmetric steering vector, then the algorithm can generate extreme values at the real and symmetric positions of the signal simultaneously. This characteristic means that the DOA estimation only needs to search half of the (u,v) domain. Therefore, the purpose of “rapidity” can be achieved.

Solving the intersection space of $U_{N}$ and $U_{N}{}^{*}$ is essential to constructing the fast search algorithm. The steps discussed below briefly describe the method to find the intersection space of the two subspaces.

First, we define a concept called “adjoint solution”. Suppose that $\left[\alpha_{1},\alpha_{2},\cdots ,\alpha_{s}\right]$ and $\left[\beta_{1},\beta_{2},\cdots ,\beta_{t}\right]$ are two groups of vectors of the linear space V, and $\left(a_{1},a_{2},\cdots ,a_{s},b_{1},b_{2},\cdots ,b_{t}\right)$ is a solution of the equation $x_{1}\alpha_{1}+x_{2}\alpha_{2}+\cdots +x_{s}\alpha_{s}=y_{1}\beta_{1}+y_{2}\beta_{2}+\cdots +y_{t}\beta_{t}$. Then, $\left(a_{1},a_{2},\cdots ,a_{s}\right)$ is called the adjoint solution of $\left(a_{1},a_{2},\cdots ,a_{s},b_{1},b_{2},\cdots ,b_{t}\right)$.

Suppose that the noise subspace $U_{N}$ is represented by $\left[\alpha_{1},\alpha_{2},\cdots ,\alpha_{M-K}\right]$ and the conjugate noise subspace $U_{N}{}^{*}$ is represented by $\left[\beta_{1},\beta_{2},\cdots ,\beta_{M-K}\right]$. Then, the intersection space $U_{inter}$ of $U_{N}$ and $U_{N}{}^{*}$ can be determined by the adjoint solution of

(13) $$x_{1}\alpha_{1}+x_{2}\alpha_{2}+\cdots +x_{s}\alpha_{s}=y_{1}\beta_{1}+y_{2}\beta_{2}+\cdots +y_{t}\beta_{t},$$

which is specifically expressed by Definition 1 in this study.

Definition 1: $U_{inter}=U_{N}\cap U_{N}{}^{*}=\left\{k_{1}\alpha_{1}+k_{2}\alpha_{2}+\cdots +k_{s}\alpha_{s}|\left(k_{1},k_{2},\cdots ,k_{s}\right)\right\}$ is the adjoint solution of the solution of Equation (13).

The definition can be proven as follows. Suppose that $\zeta =U_{inter}$. From $\zeta \in U_{N}$, we can obtain

(14) $$\zeta =k_{1}\alpha_{1}+k_{2}\alpha_{2}+\cdots +k_{s}\alpha_{s}.$$

From $\zeta \in U_{N}{}^{*}$, we can derive

(15) $$\zeta =l_{1}\beta_{1}+l_{2}\beta_{2}+\cdots +l_{s}\beta_{s}.$$

Thus, we have:

(16) $$k_{1}\alpha_{1}+k_{2}\alpha_{2}+\cdots +k_{s}\alpha_{s}=l_{1}\beta_{1}+l_{2}\beta_{2}+\cdots +l_{s}\beta_{s}.$$

Consequently, $\left(k_{1},k_{2},\cdots ,k_{s}\right)$ is an adjoint solution of Equation (13).

By contrast, if $\left(k_{1},k_{2},\cdots ,k_{s}\right)$ is the adjoint solution of a solution to that of Equation (13), then $\left(k_{1},k_{2},\cdots ,k_{s},l_{1},l_{2},\cdots ,l_{s}\right)$ is the solution of Equation (15), which is also the expression of Equation (16). Given that the left side of Equation (16) belongs to $U_{N}$, whereas the right side of Equation (16) belongs to $U_{N}{}^{*}$, we have:

(17) $$\zeta =k_{1}\alpha_{1}+k_{2}\alpha_{2}+\cdots +k_{s}\alpha_{s}\in U_{inter}.$$

In summary, $U_{inter}=U_{N}\cap U_{N}{}^{*}=\left\{k_{1}\alpha_{1}+k_{2}\alpha_{2}+\cdots +k_{s}\alpha_{s}|\left(k_{1},k_{2},\cdots ,k_{s}\right)\right\}$ is the adjoint solution of the solution of Equation (13). At this stage, the proof has been finished.

On the basis of the above algorithm, the TD spectrum is defined as

(18) $$f_{TD}\left(u,v\right)=\frac{1}{a^{H}\left(u,v\right)U_{inter}U_{inter}^{H}a\left(u,v\right)}$$

As mentioned, this spectral search function generates extreme values at the real and symmetric positions of the signal simultaneously. Thus, only a half-domain search in the (u,v) domain is needed to estimate the signal direction. Then, an fine search is performed at the vicinity of the spectral peak position and its symmetric position to obtain the precise arrival angle. Compared with the MUSIC algorithm and the RD-MUSIC algorithm proposed in reference [29], this algorithm has advantages in both speed and accuracy.

### 3.3. Description of Algorithm Steps

The implementation steps of the proposed method are summarized as follows:

Step 1: Perform eigenvalue decomposition of the array-received data matrix in order to obtain the noise subspace.

Step 2: Calculate the intersection space by the algorithm in Definition 1, and then established the TD spectrum based on Equation (18).

Step 3: Search the positive half-spectrum of Equation (17) to obtain the estimated value $\left({\hat{u}}_{i},{\hat{v}}_{i}\right)$ of the DOA parameter in the (u,v) domain, where i=1,2,⋯,K.

Step 4: Substitute $a\left({\hat{u}}_{i},{\hat{v}}_{i}\right)$ and $a\left(-{\hat{u}}_{i},-{\hat{v}}_{i}\right)$ into Equation (6) for the extreme value test. Among the two elements, the one that satisfies $a^{H}\left(u,v\right)U_{N}=0$ is the TD- DOA.

Step 5: Substitute the TD-DOA, which was obtained in step 4, into Equation (8) to calculate the spatial domain DOA $\left({\hat{\theta }}_{i},{\hat{\varphi }}_{i}\right)$.

Step 6: Perform an accurate searching in a small area near $\left({\hat{\theta }}_{i},{\hat{\varphi }}_{i}\right)$. The element that satisfies $a^{H}\left(\theta ,\varphi \right)U_{N}=0$ is the real spatial domain DOA.

As shown in these steps, the algorithm first implements a rough search process to obtain the estimated value $\left({\hat{u}}_{i},{\hat{v}}_{i}\right)$. Then, the inverse trigonometric function is transformed to determine the rough estimation of the angle $\left({\hat{\theta }}_{i},{\hat{\varphi }}_{i}\right)$. Finally, an accurate search is conducted in the small neighborhood of $\left({\hat{\theta }}_{i},{\hat{\varphi }}_{i}\right)$ to obtain the fine estimate $\left({\overset{⌢}{\theta }}_{i},{\overset{⌢}{\varphi }}_{i}\right)$. As such, no angle measurement blurring will occur.

### 3.4. Algorithm Complexity Analysis

The traditional MUSIC algorithm, the RD-MUSIC algorithm, and our algorithm are compared in this study. The array structure used by the two algorithms is shown in Figure 2. Consider that *K* uncorrelated signals impinge upon an array of *M* elements, the number of snapshots is given by *L*, and the number of search points is given by *q*.

For the traditional MUSIC algorithm, the modulus ${\left\|a^{H}\left(\theta ,\varphi \right)U_{N}\right\|}^{2}$ needs to be calculated for each spectral point, and the dimension of $U_{N}$ is M×(M−K). Therefore, the computation of the spectral search of the traditional MUSIC algorithm is q(M−K)(M+1). The computation of the eigenvalue decomposition in the M×M-dimensional auto-covariance matrix is $M{\left(K+2\right)}^{2}$. Hence, the total computation of the traditional MUSIC algorithm is $q\left(M-K\right)\left(M+1\right)+M{\left(K+2\right)}^{2}$.

For the RD-MUSIC algorithm, the computation of the eigenvalue decomposition in the M×M-dimensional auto-covariance matrix is the same as that in the MUSIC algorithm. The computation for searching the spectral peak of the reduced spectral function is q(M−K)(M/2+1).The total computation of the RD-MUSIC algorithm is $q\left(M-K\right)\left(M/2+1\right)+M{\left(K+2\right)}^{2}$.

For the algorithm proposed in this paper, the computation of the eigenvalue decomposition in the M×M-dimensional auto-covariance matrix is the same as the MUSIC algorithm. The intersection space $U_{inter}$ has a lower dimension, denoted by M×(M−2K), compared with $U_{N}$, and the search range of the TD–WNSF algorithm is reduced by half. Thus, the computation of the peak search is q(M−2K)(M+1)/2. The total computation of the TD-MUSIC algorithm is $q\left(M-2K\right)\left(M+1\right)/2+M{\left(K+2\right)}^{2}$.

Table 1 shows a comparison of the computations of the three algorithms with the number of array elements. The number of signal sources is K=2 and the search accuracy is 1°.

We can easily see that, when the number of array elements is small, the computational complexity of our algorithm is between the MUSIC algorithm and the RD-MUSIC algorithm. With the increase in the number of array elements, the computational complexity of our algorithm is far lower than that of the other two algorithms, which reflects the rapidity of our algorithm.

## 4. Experiment and Simulation Analysis

### 4.1. Parameters and Characteristics of Devices

The hardware structure of the antenna device is shown in Figure 1. In the research process, the radius of the radiation unit, the length of the microstrip line in the feed circuit, and other parameters were calculated according to theoretical knowledge [34]; then, it was optimized in the simulation software ANSSY Electronics Desktop. Finally, the optimal parameters of antenna are shown in Table 2.

The DC bias circuit is applied to control the closing or opening state of three PIN diodes (Infineon bar64-02v). The antenna can be switched between the three working states. The polarization mode and the pattern of the antenna in different working states are shown in Table 3.

**Figure 3:**
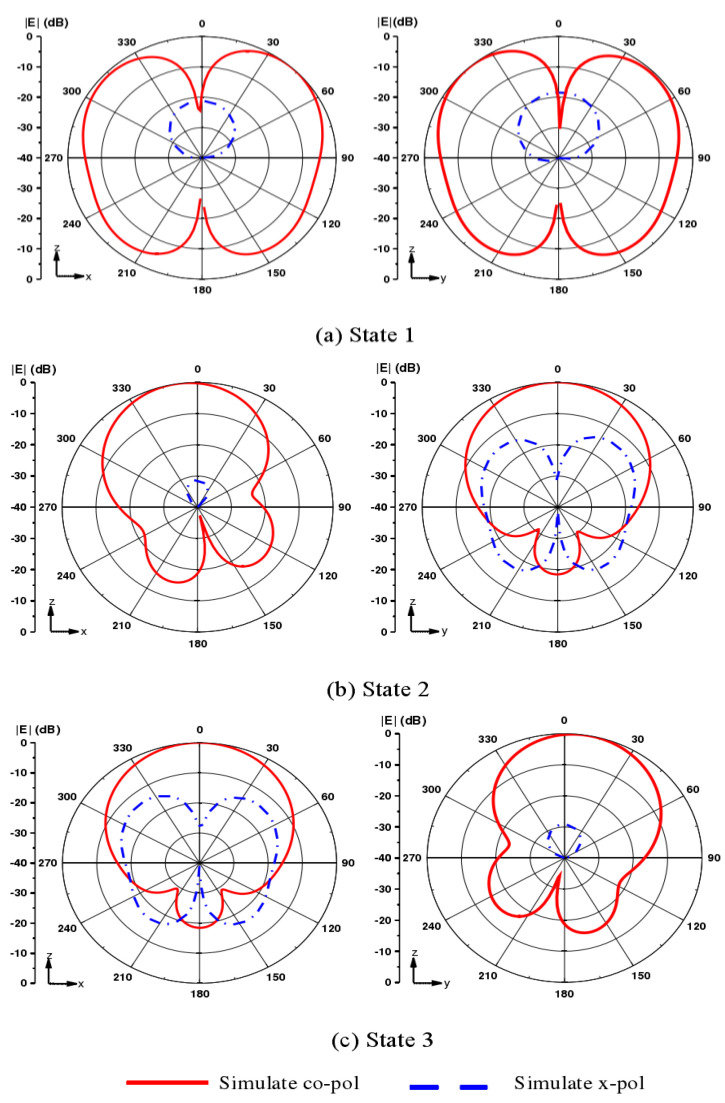
Radiation pattern in (**a**) working state 1, (**b**) working state 2, (**c**) working state 3.

Figure 3 shows the radiation pattern of the antenna in three states at 0.2 GHz. From Figure 3, it is apparent that the working mode of omnidirectional radiation is implemented in state 1, while directional radiation is implemented in state 2 and state 3. It can be seen from Figure 3b that the directional radiation pattern of the antenna is tilted, with an angle of about 8° and a gain of about 7.5 dB, which has little effect in terms of practical application.

### 4.2. Experimental Scene

**Figure 4:**
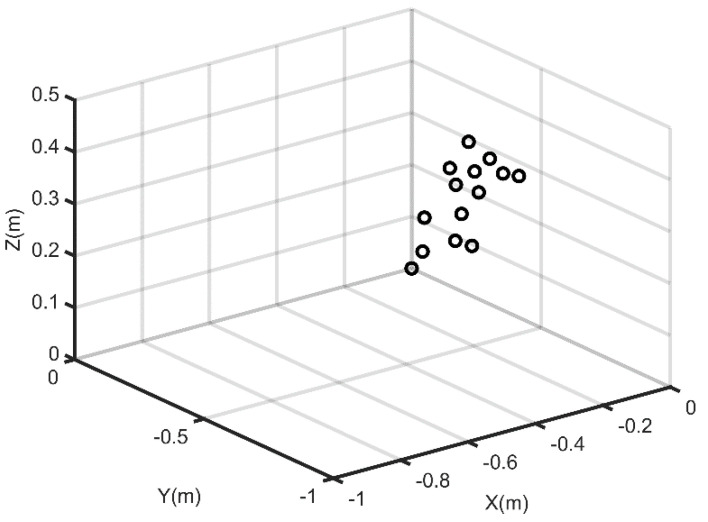
The distribution of array element position.

In this experiment, we placed the reconfigurable antenna array direction finding system into an actual environment. During the test, the UAV with a beacon and the real - time kinematic (RTK) mobile station flew at an altitude of approximately 700 m above the array area. The beacon transmitted a single tone radio frequency (RF) signal, which was received by an array of 14 antenna elements on the ground. The RTK mobile station output the coordinates of the antenna phase center of the beacon in real time. The distribution of array elements is shown in Figure 4.

**Figure 5:**
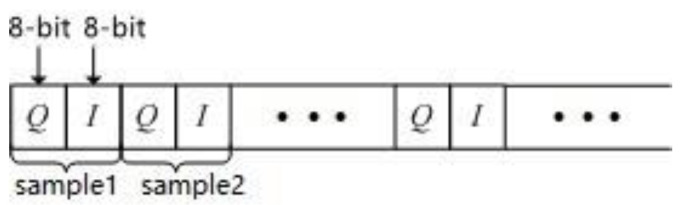
Schematic diagram of the data storage format.

The received data of the array stored in the system are baseband signals (including I-channel and Q-channel) after down-conversion. The specific format of the sampled data is shown in Figure 5. Each sample contains 8-bit Q and 8-bit I, occupying a total of 16 bits of storage space. At the same sampling point, the first part is Q and the second part is I.

### 4.3. Signal DOA Parameter Estimation

**Figure 6:**
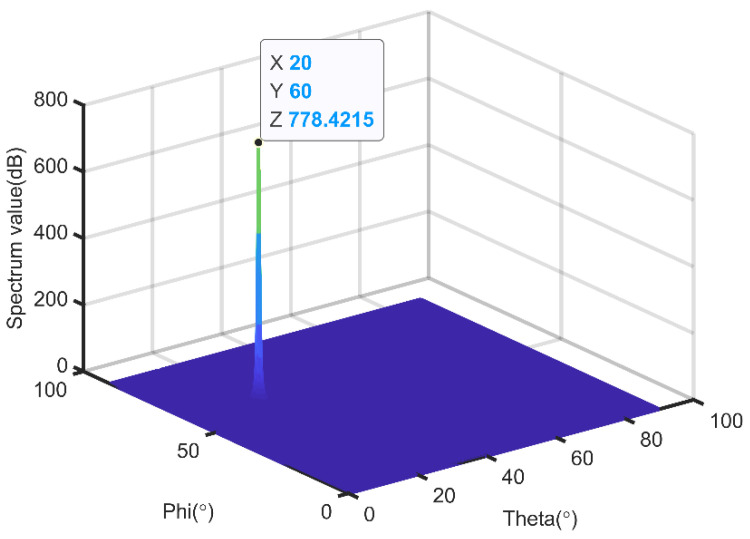
Spatial spectrum of the traditional MUSIC algorithm.

In this test, we read the baseband signal data through the computer, and then performed DOA estimation with the traditional MUSIC algorithm and our algorithm, respectively. The running result of the traditional MUSIC algorithm is shown in Figure 6.

**Figure 7:**
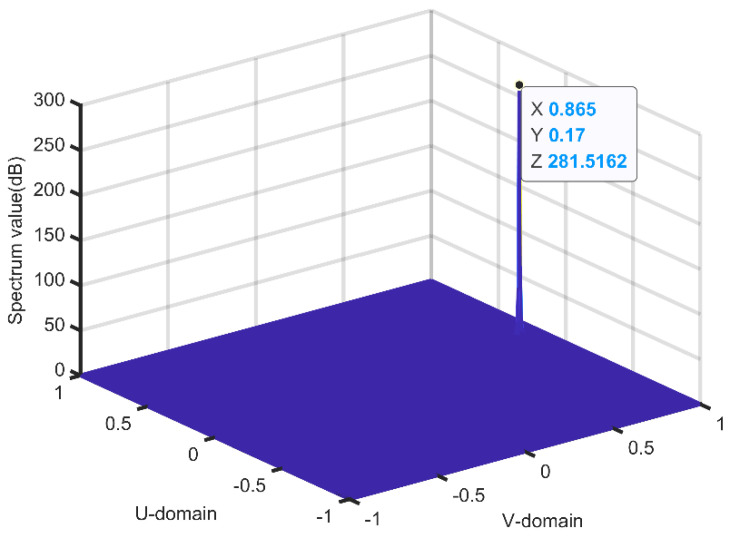
Transform domain spectrum of our algorithm.

From Figure 6, we can see that the spatial DOA of the incoming wave is (60°, 20°). The running result of our algorithm is shown in Figure 7. From Figure 7, we can see that the estimation of TD-DOA is (0.17, 0.865). According to Equation (8), the spatial angle (60°, 20°) and the TD angle (0.17, 0.865) are equivalent, which illustrates that our algorithm can accurately measure the DOA of the signal.

### 4.4. Relationship Between Algorithm Performance and SNR

**Figure 8:**
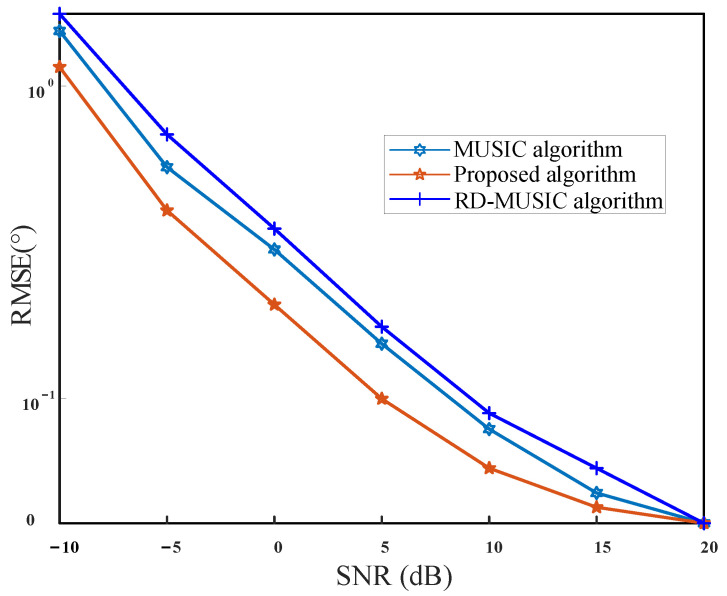
RMSE versus the SNR for theta.

**Figure 9:**
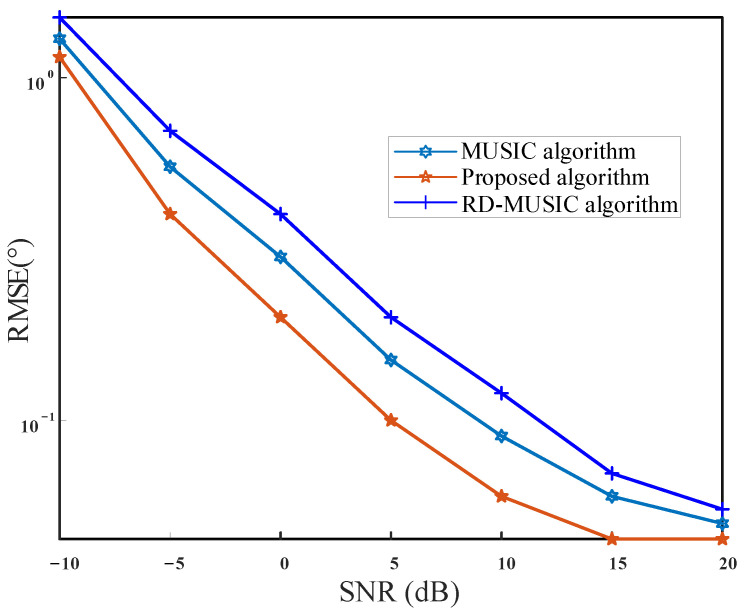
RMSE versus the SNR for phi.

A simulation was implemented to compare the DOA estimation performances of the MUSIC algorithm, the RD-MUSIC algorithm, and the proposed algorithm. We considered two incoherent signals to be incidental to the array. The theta angle is $\theta =\left[70^{o},30^{o}\right]$, the phi angle is $\varphi =\left[10^{o},50^{o}\right]$, and the corresponding TD angle is u=[0.9254,0.3214], v=[0.1632,0.383]. The Monte Carlo number is L= 500 and the SNR shifts from −10 to 20 dB. The root-mean-squared error (RMSE) was used to measure the effectiveness of these three algorithms, which was defined as

(19) $$\left\{ \begin{array}{l} RMSE_{\theta }=\frac{1}{K}\sum_{k=1}^{K}\sqrt{\frac{1}{L}\sum_{l=1}^{L}{\left({\hat{\theta }}_{k,l}-\theta_{k}\right)}^{2}}\\ RMSE_{\varphi }=\frac{1}{K}\sum_{k=1}^{K}\sqrt{\frac{1}{L}\sum_{l=1}^{L}{\left({\hat{\varphi }}_{k,l}-\varphi_{k}\right)}^{2}} \end{array}\right.,$$

where ${\hat{\theta }}_{k,l}$ and ${\hat{\varphi }}_{k,l}$ represent the estimated values of the kth signal in the lth Monte Carlo simulation, respectively. Figure 8 shows the relationship between the RMSE of the theta angle and SNR, while Figure 9 shows the relationship between the RMSE of the phi angle and SNR.

From Figure 8 and Figure 9, we can see that the RMSE curves of three methods decrease as SNR increases. However, the RMSE curve of our method locates below that of MUSIC and RD-MUSIC all along, which indicates that our method is superior to MUSIC and RD-MUSIC under different SNRs. This is because our method performs many fine-grained searches in the neighborhood of the TD estimation.

### 4.5. Relationship Between Algorithm Performance and Snapshots

**Figure 10:**
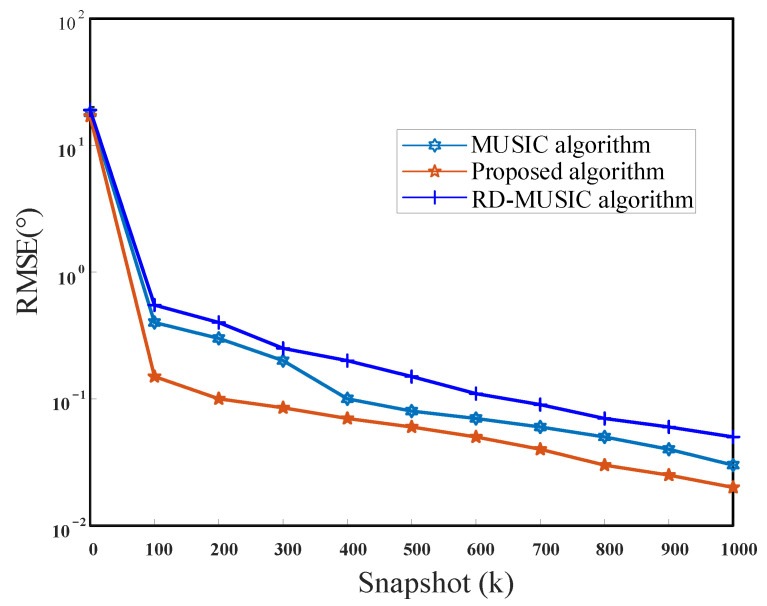
RMSE versus the snapshot for theta.

**Figure 11:**
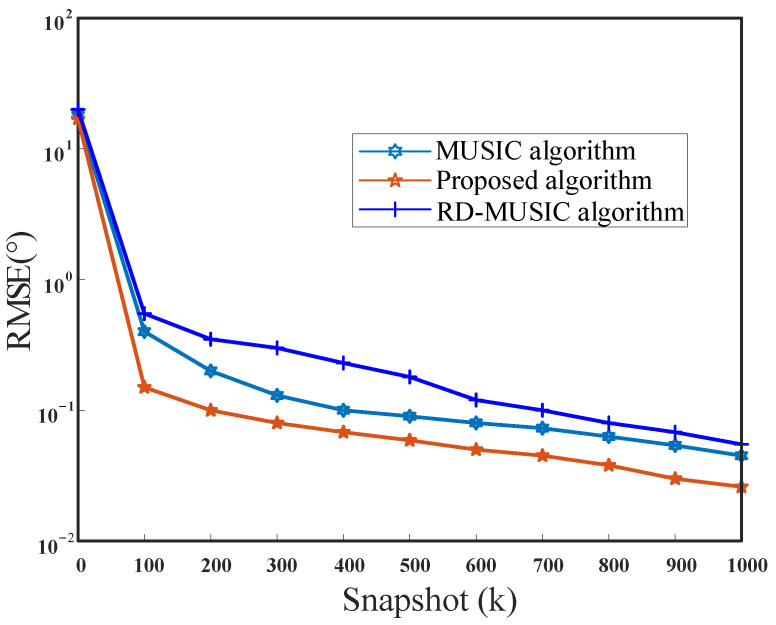
RMSE versus the snapshot for phi.

To observe the performance of the new approach more clearly, we compared the relationship between algorithm performance and snapshots. In this simulation, the number of snapshots varied over a wide range, from 5 to 1000. The number of signals is K=2 and SNR=10 dB. Figure 10 and Figure 11 show the curves of RMSEs of the theta angle and the phi angle change with the snapshots, respectively. When the SNR is the same, the RMSE of the DOA estimates decreases with the increase in the number of snapshots. The proposed method performs better than MUSIC and RD-MUSIC, especially in the case of small snapshots.

## 5. Conclusions

In this paper, a reconfigurable antenna array direction finding system was proposed. This system can dynamically control the working state of each antenna and determine the number of working antennas according to the number of signal sources. The system consists of two parts, namely, the hardware structure of a configurable antenna and an improved direction-finding algorithm. The hardware of the reconfigurable antenna is a double-layer structure, while the algorithm converts the DOA parameters of the traditional angle domain to the transform domain. Compared with the traditional MUSIC algorithm and the RD-MUSIC algorithm, the computation of the proposed algorithm is significantly reduced. The actual test results show that the system can accurately estimate the DOA of the incident signal. The simulation results indicate that the proposed algorithm has high accuracy and efficiency.

## Figures and Tables

**Table 1:** Computation of the two algorithms.

Array Number	5	6	7	8	9	10
MUSIC	3320	5136	6952	9848	12,744	16,000
RD-MUSIC	1970	2976	4162	5528	7074	8800
Our algorithm	3120	3876	4432	4988	5544	6100

**Table 2:** Parameters of reconfigurable antenna.

Parameter	Value (mm)	Parameter	Value (mm)
$R_{1}$	10	$W_{2}$	1.4
$R_{2}$	24.5	l	13
p	1	H	8
$W_{1}$	5	$R_{g}$	70

**Table 3:** Relationship between working state of antenna and diode.

Working State	Switch 1	Switch 2	Switch 3	Polarization	Pattern
State 1	off	off	on	Vertical	Omnidirectional
State 2	off	on	off	X-horizontal	Directional
State 3	on	off	off	Y-horizontal	Directional

## Data Availability

The data that support the findings of this study are available from the corresponding author upon reasonable request.
